# A patient-derived organoid-based study identified an ASO targeting SNORD14E for endometrial cancer through reducing aberrant FOXM1 Expression and β-catenin nuclear accumulation

**DOI:** 10.1186/s13046-023-02801-2

**Published:** 2023-09-05

**Authors:** Xi Chen, Xin Liu, Qian-hui Li, Bing-feng Lu, Bu-min Xie, Yu-meng Ji, Yang Zhao

**Affiliations:** Department of Obstetrics and Gynecology, Department of Gynecologic Oncology Research Office, Guangzhou Key Laboratory of Targeted Therapy for Gynecologic Oncology, Guangdong Provincial Key Laboratory of Major Obstetric Diseases, The Third Affiliated Hospital of Guangzhou Medical University, No.63 Duobao Road, Liwan District, Guangzhou, 510150 Guangdong Province PR China

**Keywords:** Endometrial cancer, Patients-derived organoid, ASO targeted therapy, Alternative splicing, 2’-O-methylation modification

## Abstract

**Background:**

Most of the endometrial cancer (EC) patients are diagnosis in early stage with a good prognosis while the patients with locally advanced recurrent or metastatic result in a poor prognosis. Adjuvant therapy could benefit the prognosis of patients with high-risk factors. Unfortunately, the molecular classification of great prognostic value has not yet reached an agreement and need to be further refined. The present study aims to identify new targets that have prognostic value in EC based on the method of EC patient-derived organ-like organs (PDOs), and further investigate their efficacy and mechanism.

**Methods:**

The Cancer Genome Atlas (TCGA) database was used to determine SNORD14E expression. The effects of SNORD14E were investigated using CCK8, Transwell, wound-healing assays, and a xenograft model experiment; apoptosis was measured by flow cytometry. Antisense oligonucleotide (ASO) targeting SNORD14E was designed and patient-derived organoids (PDO) models in EC patients was established. A xenograft mouse and PDO model were employed to evaluate the effects of ASO targeting SNORD14E. RNA-seq, Nm-seq, and RNA immunoprecipitation (RIP) experiments were employed to confirm the alternative splicing (AS) and modification induced by SNORD14E. A minigene reporter gene assay was conducted to confirm AS and splicing factors on a variable exon. Actinomycin-d (Act-D) and Reverse Transcription at Low deoxy-ribonucleoside triphosphate concentrations followed by PCR (RTL-P) were utilized to confirm the effects of 2′-O methylation modification on FOXM1.

**Results:**

We found that SNORD14E was overexpressed in EC tissues and patients with high expressed SNORD14E were distributed in the TCGA biomolecular classification subgroups without difference. Further, SNORD14E could reduce disease-free survival (DFS) and recurrence free survival (RFS) of EC patients. SNORD14E promoted proliferation, migration, and invasion and inhibited the apoptosis of EC cells in vitro. ASOs targeting SNORD14E inhibited cell proliferation, migration, invasion while promoted cell apoptosis. ASOs targeting SNORD14E inhibited tumor growth in the xenograft mouse model. TCGA-UCEC database showed that the proportion of patients with high expression of SNORD14E in middle-high risk and high-risk patients recommended by EMSO-ESGO-ESTRO guidelines for adjuvant therapy is more than 50%. Next, we enrolled 8 cases of high-risk and high-risk EC patients according to EMSO-ESGO-ESTRO guidelines and successfully constructed EC-PDOs. ASOs targeting SNORD14E inhibited the EC-PDO growth. Mechanistically, SNORD14E could recognize the mRNA of FOXM1 and recruit SRSF1 to promote the shearing of the variable exon VIIa of FOXM1, resulting in the overexpression of the FOXM1 malignant subtypes FOXM1b and FOXM1c. In addition, SNORD14E modified FOXM1 mRNA with 2`-O-methylation, which prolonged the half-life of FOXM1 mRNA. The nucleus accumulation of β-catenin caused by aberrant FOXM1 expression led to EC progression.

**Conclusions:**

ASO targeting SNORD14E can be an effective treatment for EC.

**Supplementary Information:**

The online version contains supplementary material available at 10.1186/s13046-023-02801-2.

## Background

Endometrial cancer (EC) is one of the most common gynecological malignancy in women. Most of the EC patients were diagnosed in the early stage. About 80% patients with early EC had a good prognosis, and the 5-year overall survival rate was 95% [1, 2]. The prognosis of patients with advanced and recurrent EC is poor (5-year survival rate < 20%) [3]. Although the risk of recurrence of early EC after initial treatment is low (7.2%), about 15–20% EC patients may have a high risk of advanced disease or disease recurrence, and the recurrence rate is close to 20–25% [4, 5]. Identifying and giving high-risk patients the best adjuvant therapy to improve their prognosis is still the biggest challenge of EC treatment.

In 2013, TCGA first proposed four distinct molecular classification based on the molecular characterization of EC [6]. Recently, new molecular classification methods such as ProMisE, TransPORTEC and Parra-Herran have been proposed due to the complicated and poor in clinical translation of the original TCGA molecular classification. At present, all biomolecular classification methods still have inevitable errors, and no consistent conclusion has been reached so far [7–9].

The ESMO-ESGO-ESTRO guidelines recommend adjuvant therapy for patients in middle-high risk group and high-risk group to improve prognosis [3]. We take snoRNA as the research target and analyze the TCGA-UCEC database. We have found that SNORD14E was aberrantly expressed in EC tissues and reduce DFS and RFS of patients with EC. The patients with high expressed SNORD14E were distributed in the TCGA biomolecular classification subgroups without difference. The results suggested that SNORD14E could be a target that can affect the prognosis of EC patients independent of biomolecular classification. We further grouped the EC patients in TCGA database based on the ESMO-ESGO-ESTRO risk classification, and found that patients with high level of SNORD14E were widely presented in all risk subgroup. Notably, the proportion of patients with high level of SNORD14E in the middle-high risk group and high-risk group which recommended by the guidelines for adjuvant therapy are over 50%. This suggested that the treatment targeting SNORD14E may bring benefits to the prognosis of nearly 50% patients in the middle-high risk group and high-risk group. The following in vitro experiments in EC cells showed that SNORD14E promoted proliferation, migration, and invasion and inhibited the apoptosis. ASOs targeting SNORD14E inhibited cell proliferation, migration, invasion while promoted cell apoptosis. ASOs targeting SNORD14E inhibited tumor growth in the xenograft mouse model.

Due to the complexity of human tumors, the response to clinical cancer treatments varies substantially. Recently, organoid culture technologies have been developed, including EC tissues [10]. Patient-derived organoids (PDOs) maintains pathological and molecular properties of EC with great experimental accessibility and lower cost than animal models. Additionally, PDOs are considered to better represent the natural state of the tumor than cell lines and therefore serves as the suitable models for potential treatment reagents. In order to further explore the therapeutic effect of ASO targeting SNORD14E, we constructed 8 EC-PDO models from patients with high level of SNORD14E in middle-high risk or high-risk group, and found that ASO-SNORD14E can inhibit the proliferation of the 8 EC-PDOs. In this study, we have identified a snoRNA, SNORD14E, which plays an oncogenic role in EC via two distinct mechanisms to induce β-catenin nuclear accumulation through FOXM1. The therapeutic value of the targeted drug ASO-SNORD14E was verified at multiple levels, including the EC-PDOs. We have further investigated the molecular mechanism of malignant biological behavior in EC caused by SNORD14E, and the potential of SNORD14E as a therapeutic target to improve the prognosis of patients with EC, especially those in middle-high risk and high-risk group.

## Methods

### Study design

The study is a preclinical study which aims to assess the therapeutic effect of the ASO-SNORD14E in EC patients at multiple levels. The participants were required to provide written informed consent before participating in the study. The study protocol was approved by the Ethics Committee of Guangzhou Medical University (2020066). Patients received standard treatment according to clinical treatment guidelines, and patient tissues obtained from surgery were used to construct PDO and evaluate the responsiveness to ASO (Fig. 8F).

### Setting and participants

Participants were adults (age ≥ 18 years old) histologically confirmed EC, and none of the enrolled patients received anti-tumor related treatment. The participants were all from patients with EC who underwent surgery in the Third Affiliated Hospital of Guangzhou Medical University from April 22, 2022 to March 30, 2023. And all the patients were operable endometrial cancer patients. Risk classification is carried out according to ESMO-ESGO-ESTRO guideline. The expression of SNORD14E of EC patients in middle-high risk group and high-risk group was detected, and patients with high expression of SNORD14E were selected for EC-PDO construction and ASO treatment (Fig. 8G, Supplementary Table 4). The 8 patients enrolled in the study were all survival without recurrence due to the short postoperative time.

### Variables

In the study, self-control was adopted to minimize confounder factors as EC-PDOs were from tissues of EC patients. EC-PDOs from the same EC patient tissue were randomly grouped and treated with ASO drugs or untreated. The changes of EC-PDO volume were counted respectively, and the proliferation of PDO was measured by CellCounting-Lite 3D luminescent cell vitality.

### Bias

Firstly, considering the selection bias and confounder bias caused by the difference of individual responses to drugs, we have PDOs from the same patient were randomly divided into experimental group and control group for treatment respectively. Secondly, in order to avoid the selection bias caused by the PDO initial volume, PDOs with the initial diameter limit of 50–150 μm [11] were selected for tracking and statistical analysis. Thirdly, there may be information bias caused by the difference between the actual size of PDO and the measured value due to the irregular shape of PDOs. We use the change of PDO area to measure the effect of ASO drugs on PDO proliferation. Fourth, different researchers are responsible for grouping and measurment separately to collect data blindly to limit information bias. Finally, in order to avoid the confounder bias caused by pollution, growth environment differences and other factors, medium both of the experimental group and the control group were changed and added at the same time during the treatment, and keeping asepsis.

### Study size

Eight samples that can meet the enrollment criteria were included in the study to construct PDO. The PDOs with appropriate size (diameter 50-150 μm) were selected for statistical analysis to avoid the influence of PDO initial volume. In the eight cases of EC-PDO, no less than 5 PDOs of each case were included in each group for measurement, and the data conform to normal distribution and statistically analyzed by paired *t*-test.

### PDO models and ASO treatment

The generation of EC organoids was performed as described previously [12]. Briefly, EC tissues were cut into 1–3-mm^3^ pieces and digested for 15 min in TrypLE (12604013, Thermo Fisher Scientific). The organoid was suspended with 40% complete medium [13] and 60% Matrigel mixture. Then, 20-µL droplets were deposited in prewarmed 48-well plates (Corning dishes). Passaging was performed every 10–15 days.

For transfection-free uptake in ASO treatment, 8 nM SNORD14E-specific ASO or ASO-NC was added into medium (Supplementary Table 3). An organoid and ASO were co-incubated for 7 or 9 days. For visualization of organoid proliferation, images were obtained using an inverted microscope (Nikon) at 4 × magnification.

### Organoid lentiviral transduction

Organoids were digested and resuspended in a medium. Cells were spin-infected (700 g, 90 min, 25 °C) on low-adhesion plates (Corning), and incubated at 37 °C for 4–5 h. The organoids were then centrifuged at 300 g for 3 min at 4 °C and seeded in Matrigel.

### PDO viability assay

The viability of organoids was measured following the methods of CellCounting-Lite 3D Luminescent Cell Viability Assay (Vazyme, DD1102-01). Briefly, organoids were digested and seeded into 96-well plates. Each plate was placed at room temperature for 30 min before adding an equal volume of CellCounting-Lite 3D, followed by vigorous shaking for 5 min to lyse the cell mass fully. Plates were incubated at 25 °C for 25 min and the fluorescence intensity was detected.

### Human EC xenograft tumor models and treatment

All animal experiments were approved by the Animal Experimental Ethics Committee of Guangdong Medical Laboratory Animal Center. Briefly, 1 × 10^6^ HEC-1B cells and 2 × 10^6^ Ishikawa cells were injected subcutaneously into 4–6-week-old female nude mice. The tumor were monitored every three days for 40 days. After successful formation, tumors were collected for further IHC staining. Tumor volumes were measured and calculated as: Tumor volume (mm^3^) = 0.5 × length × width^2^. Tumor diameters were measured with a Vernier caliper.

ASO treatment of the xenograft tumor was initiated after the tumor volume reached 50mm^3^. Mice were randomized (five mice with HEC-1B cells per group and four mice with Ishikawa cells per group), and ASO-NC (8 nM) and ASO-SNORD14E (8 nM) were separately injected into tumors. The diameters of the tumors were monitored every three days, and the mice were sacrificed approximately 40 days after cell injection.

### Immunohistochemistry (IHC) and immunofluorescence (IF) assays

Tissues were fixed with 4% paraformaldehyde at 4 °C, while PDOs were fixed with 4% paraformaldehyde at room temperature for 15 min. Samples were embedded in paraffin. Then, the 5-μm-thick slices were subjected to IHC or IF assays. The antibodies used for staining were: ER (Proteintech, 21244–1-AP), PR (Proteintech, 25871–1-AP), and Ki67 (Proteintech, 27309–1-AP), FOXM1 (Proteintech, 13147–1-AP).

### Cell culture

The human EC cell lines HEC-1A, HEC-1B, Ishikawa, and KLE were purchased from the American Type Culture Collection (ATCC), and the hESC, hEEC and 293 T cell lines were obtained from Guangzhou Jennio Biotech Co., Ltd (Guangzhou, China). Cells were cultured by Dulbecco’s Modified Eagle Medium (HEC-1B) or RPMI 1640 medium (Ishikawa) with 10% FBS and 1% penicillin/streptomycin.

### Cell proliferation, migration, and invasion assays

Cell growth was detected using CCK-8 assays. The ability of migration and invasion were assessed by scratch assays and Transwell invasion assays as earlier reported [14].

### RNA-seq and Nm-seq

HEC-1B cells transfected with vector or SNORD14E plasmids were harvested. Total RNA was obtained using TRIzol reagent (Takara) as described before and sent for sequencing. NM-Seq service was provided by CloudSeq Biotech Inc. (Shanghai, China) by following the published procedures [15]. Libraries were constructed using NEBNext Small RNA Library Prep Set for Illumina (New England BioLabs). Sequencing was then performed on Illumina HiSeq4000 according to the manufacturer’s instructions.

### Reverse transcription-polymerase chain reaction (RT-PCR) analysis of splicing isoforms

Total RNA was isolated using the TRIzol reagent (Takara) following the standard protocol. qRT-PCR was performed according to the instructions of Hieff® qPCR SYBR Green Master Mix (High Rox Plus) (Yeasen, Shanghai, China). The RNA changes were calculated by the 2^−△△Ct^ method. Primers were synthesized by BGI company (Shenzhen, China) (Supplementary Table 1).

### RNA Immunoprecipitation (RIP)

Briefly, cells were lysed with RIP lysis buffer containing an RNA inhibitor and protease. The supernatant of the cell lysate was collected and incubated with antibody-conjugated beads at 4 °C overnight. The binding complexes were washed, purified, and analyzed by qRT-PCR.

### Minigene assay

FOXM1 minigene plasmids were constructed based on the genomic sequence spanning exons VIIa of the human FOXM1 gene and cloning it into the GV658 vector. Mutants with a specific sequence deletion were created based on the primary minigene.

### Nuclear-cytoplasmic fractionation

To perform subcellular fractionation of RNA and protein, we followed the instructions on the Nuclear and Cytoplasmic Protein Extraction Kit (Beyotime, P0028). Then, we added 1 mM PMSF (Beyotime, ST506) to the nuclear and cytoplasmic fractions. RNA was extracted using the TRIzol protocol (Takara, 9108). Protein was collected and target protein was detected with western-blot.

### Western-blot analysis

Proteins were extracted with RIPA lysate (#P0013B, Beyotime, Shanghai, China) or Nuclear and Cytoplasmic Protein Extraction Kit (Beyotime, P0028) and measured using BCA protein assay kit (#P0009, Beyotime, Shanghai, China). Protein (30ug) was pipetted into SDS-PAGE gel and transferred to PVDF membrane. Membranes were blocked and incubated with primary and secondary antibodies. Imaging was performed using BeyoECL Star (#P0018AM, Beyotime, Shanghai, China).

### RTL-P assay for RNA 2′-O-methylation

100 ng of total RNA was used for RT reacted in 25-μL reaction cocktails with and 50 μM of specific RT primers (Supplementary Table 2) that were denatured at 70 °C for 10 min, and then placed on ice. The RT buffer containing 200 U M-MLV reverse transcriptase (Takara), 40 U RNasin Ribonuclease inhibitor (Takara), and a low (10 μM) or high (1 mM) concentration of dNTPs were employed for an initial annealing step at 42 °C for 1 h and heated at 70 °C for 15 min. Then, the PCR reaction was terminated.

### RNA stability assay

EC cells were transfected with SNORD14E or a vector and treated with actinomycin D (ActD, S8964, Selleck) at a final concentration of 50 μg/mL for 2, 4, 6, 8, and 10 h. Total RNA was extracted and analyzed by qRT-PCR. Then, a calculation of the RNA half-life of SNORD14E was performed.

### Statistical analysis

GraphPad Prism 8.0 (La Jolla, CA, USA) was employed for statistical analyses and ImageJ measures PDO area. Volcano plot was plotted by https://www.bioinformatics.com.cn, an online platform for data analysis and visualization. All experiments were performed at least three times. For two-group comparisons, we employed the *t*-test, whereas one- or two-way ANOVA was conducted for multiple comparisons with 95% confidence intervals. *P* < 0.05 (*), *P* < 0.01 (**), and *P* < 0.001 (***).

## Results

### SNORD14E is overexpressed in endometrial carcinoma and is associated with poor prognosis

**Fig. 1:**
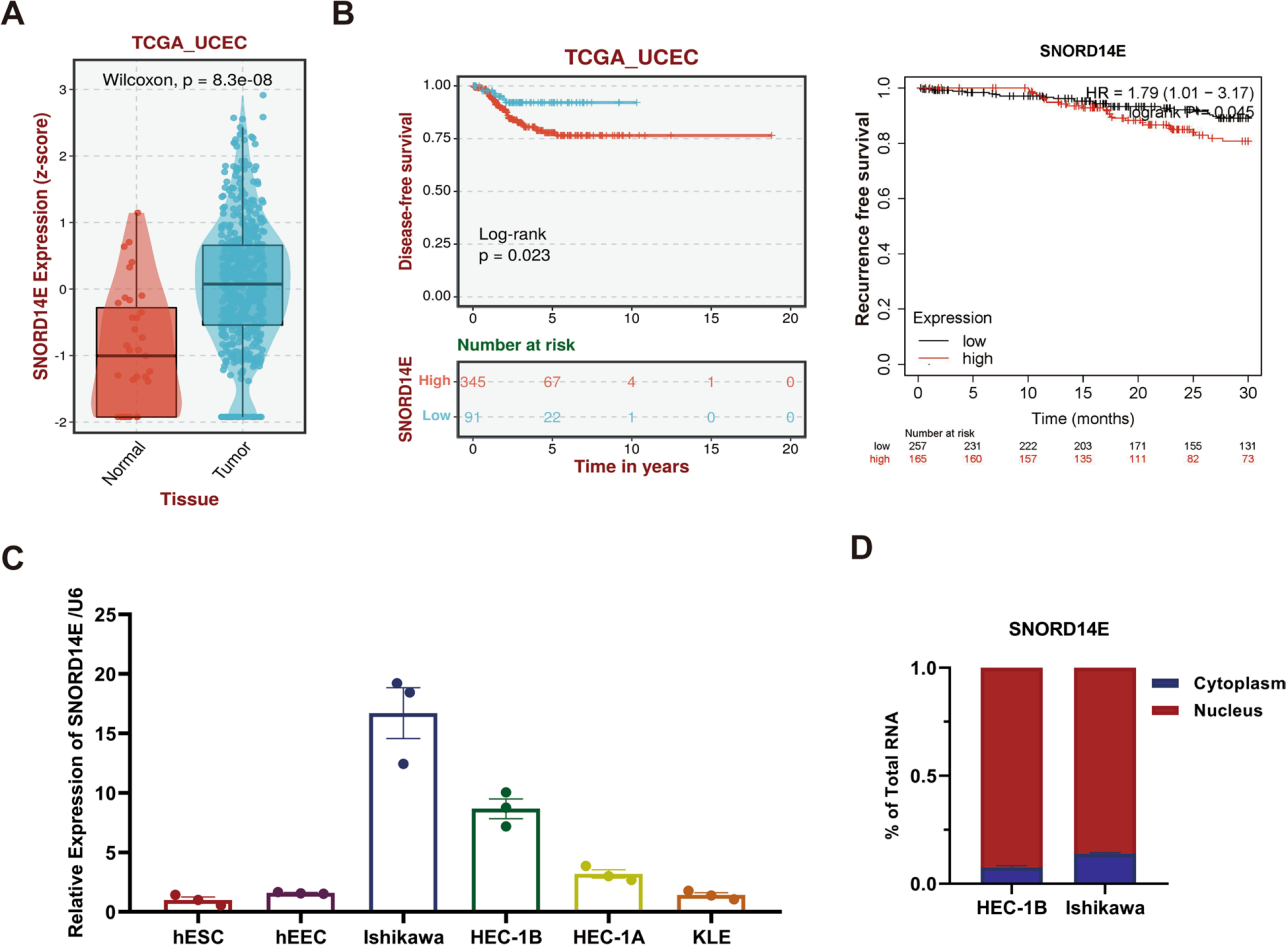
SNORD14E is overexpressed in endometrial carcinoma and is associated with poor prognosis. **A** The expression of SNORD14E in normal endometrial tissue and in endometrial cancer tissue from the TCGA dataset. **B** Kaplan–Meier analysis of Disease-free survival (*P* = 0.023, log-rank test) and Recurrence free survival (*P* = 0.045) of EC patients with low or high SNORD14E expression according to the TCGA dataset. **C** The relative expression level of SNORD14E in EC cell lines, including HEC-1A, HEC-1B, Ishikawa and KLE examined by Real-time RT-PCR, compared to normal human endometrium cell line: hESC and hEEC. **D** SNORD14E was majorly localized in the nuclear of HEC-1B cells using nuclear and cytoplasmic RNA fractionation assay followed by Real-time RT-PCR

To investigate the role of SNORD14E in EC, we analyzed a UCEC dataset from The Cancer Genome Atlas (TCGA, http://cancergenome.nih.gov) and found that SNORD14E was upregulated in EC (Fig. 1A). The characteristics of patients with EC were collected from TCGA database. The patients were divided into two groups (high vs. low) based on the the optimal critical value of SNORD14E in TCGA-UCEC selected by Kaplan–Meier, a prognostic analysis website (http://kmplot.com/analysis/) [16]. It showed that patients with high expressed SNORD14E were distributed in the TCGA biomolecular classification subgroups without difference, and the overexpression of SNORD14E was correlated with reduced DFS and RFS (Fig. 1B). The prognosis risk group of TCGA-UCEC patients according to ESMO-ESGO-ESTRO risk classification showed that the proportion of patients with high level of SNORD14E in the middle-high risk group and the high-risk group was more than 50%.

Finally, we examined SNORD14E expression in EC cell lines, which included HEC-1A, HEC-1B, KLE, Ishikawa, and normal endometrial cell lines hEEC and hESC. Our results showed that SNORD14E was more upregulated in EC cells than in normal endometrial cell lines (Fig. 1C). Nuclear/cytoplasm fractionation analysis revealed that SNORD14E was located in both the cytoplasm and the nucleus (Fig. 1D). These data suggest that SNORD14E may be involved in EC progression.

### SNORD14E promotes malignant biological behavior of EC cells in vitro and in vivo

**Fig. 2:**
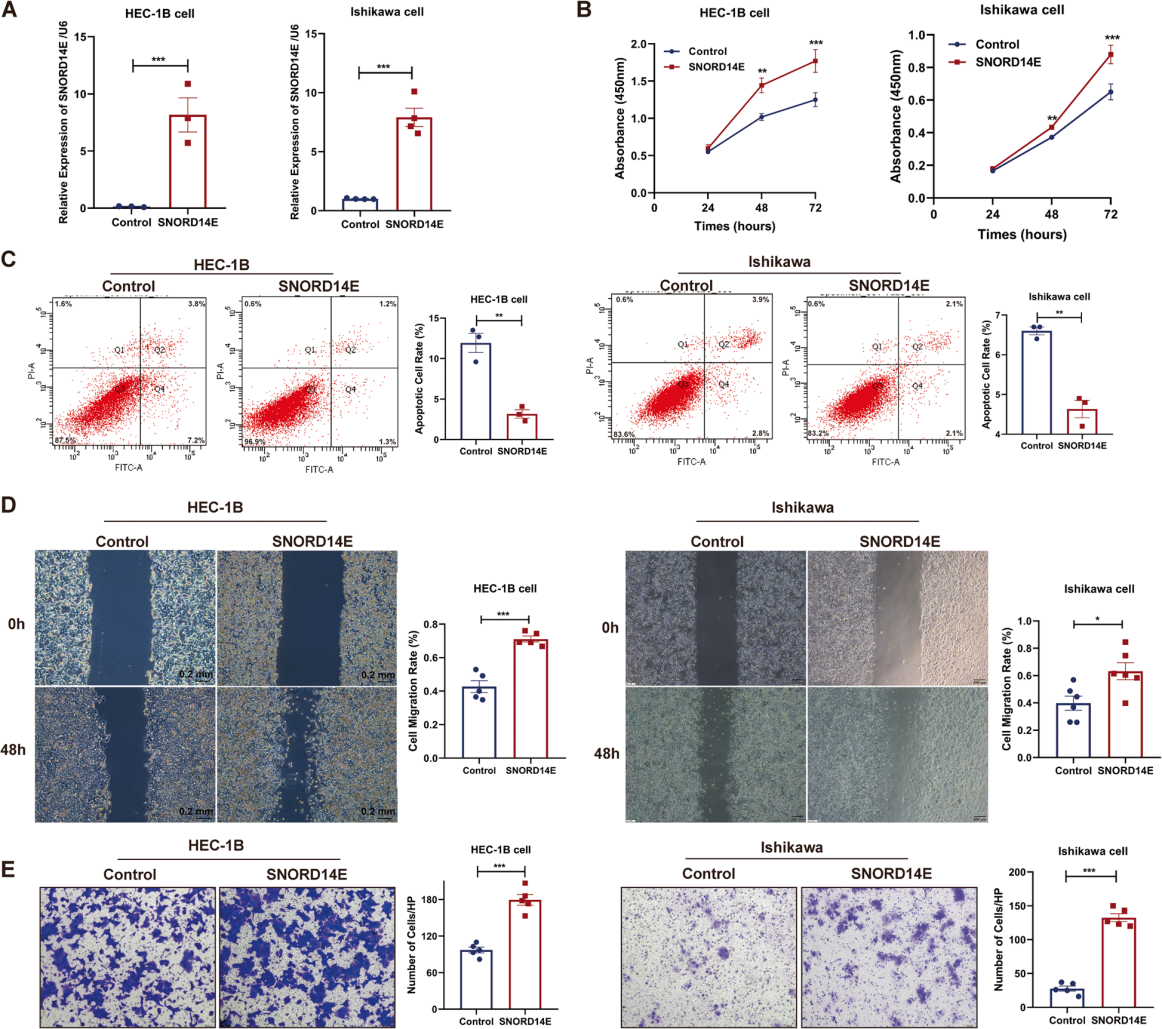
SNORD14E promotes malignant biological behavior of endometrial carcinoma cells in vitro and in vivo. **A** RT-PCR analysis was performed to detect the RNA levels of SNORD14E in HEC-1B and Ishikawa cells with or without SNORD14E stable overexpression. **B** Cell proliferation assays of HEC-1B and Ishikawa cells with or without SNORD14E overexpression. **C** Flow apoptosis was performed to analyze cell apoptosis caused by SNORD14E overexpression. Wound healing and Transwell assays (**D-E**) were employed for detecting cell migration and invasion caused by SNORD14E. * *P* < 0.05, ** *P* < 0.01, *** *P* < 0.001

Considering that SNORD14E is potentially involved in EC progression, we constructed SNORD14E-expressing plasmids and transfected them into Ishikawa and HEC-1B cell lines (Fig. 2A). Our results showed that SNORD14E promoted cell proliferation (Fig. 2B). Less apoptosis was observed in cells with upregulated SNORD14E (Fig. 2C). Compared with the control group, SNORD14E significantly promoted cell migration and invasion (Fig. 2D–E). Collectively, SNORD14E plays a crucial role in EC in vitro.

### SNORD14E recruits SRSF1 and affects FOXM1 alternative splicing

**Fig. 3:**
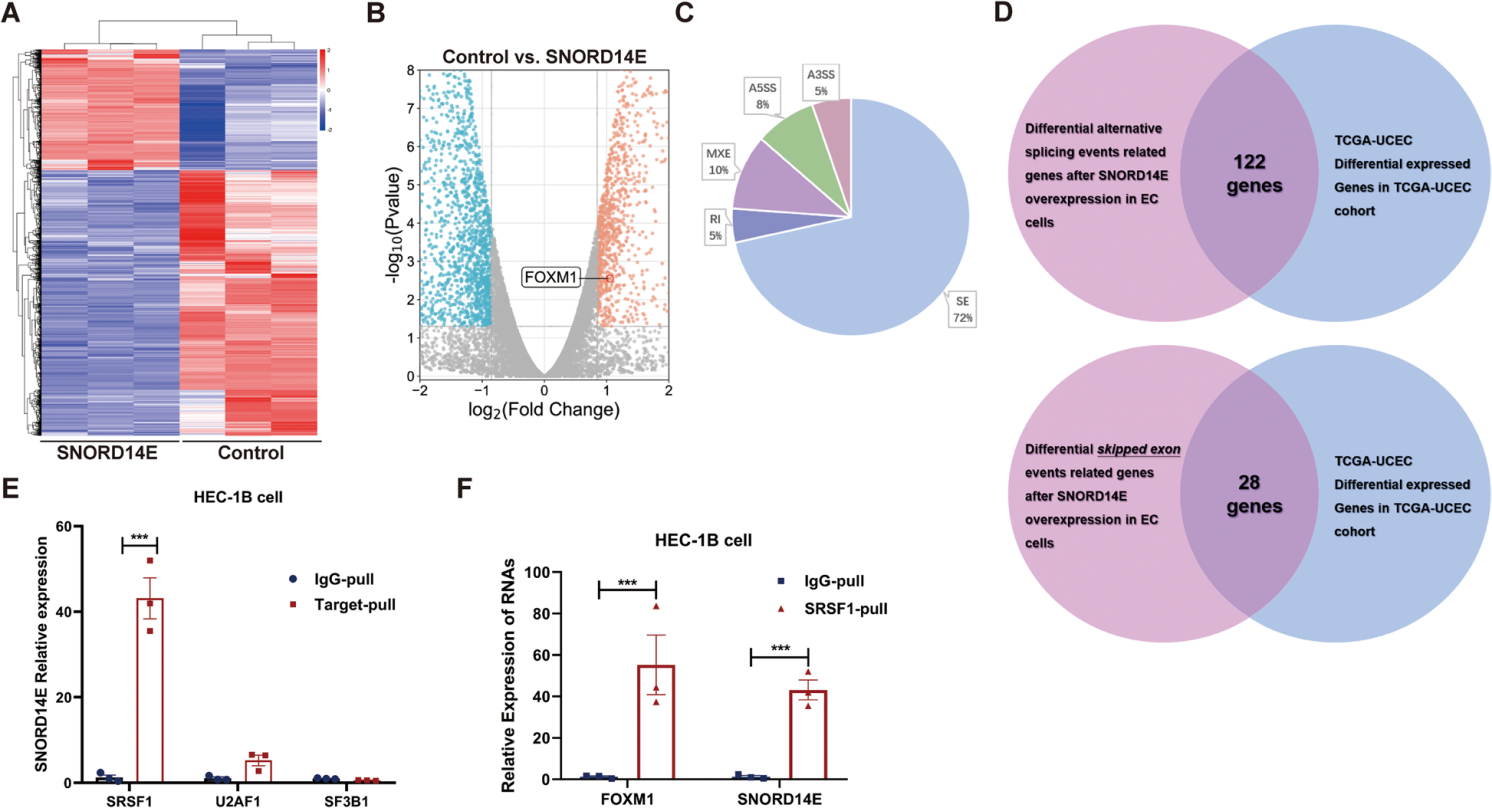
SNORD14E recruits SRSF1 and affects FOXM1 alternative splicing. **A** Heatmap of differentially expressed mRNAs in RNA-seq of HEC-1B cells with or without SNORD14E overexpression. **B** Volcano plot of differentially expressed genes between SNORD14E and control. Red dots represent upregulated genes, blue dots represent downregulated genes, and gray dots represent genes that were not differentially expressed. **C** AS events following SNORD14E overexpression. SE event accounts for the most (75%). **D** Venn diagram showing significantly differential expressing genes in TCGA-UCEC cohort and differential AS events caused by SNORD14E in RNA-seq. **E** RT-qPCR of RNA immunoprecipitation (RIP) assay for detecting the enrichment of SNORD14E on AS factors SRSF1, U2AF1, and SF3B1. IgG was used as negative control. **F** RT-qPCR of the anti-SRSF1 RIP assay showed the interaction between SRSF1-SNORD14E complex and FOXM1 mRNA. * *P* < 0.05, ** *P* < 0.01, *** *P* < 0.001. Skipped exon (SE), retained intron (RI), alternative 5’ splice site (A5SS), alternative 3′ splice site (A3SS), mutually exclusive exon (MXE)

To establish the mechanisms by which SNORD14E promotes EC progression, we conducted RNA-seq of HEC-1B cells with and without SNORD14E overexpression. The differentially expressed genes heatmaps and volcano plots are represented in Fig. 3A–B. Interestingly, differential AS events occurred after SNORD14E overexpression, including 852 skipped exons (Fig. 3C). We identified 122 genes that were differentially expressed in TCGA-UCEC and involved in the differential AS events. Additionally, 28 critical genes were identified to be both differentially expressed in TCGA UCEC and involved in skipped exon events after SNORD14E overexpression (Fig. 3D).

Studies have shown that over 300 splicing factors can regulate AS [17–19]. Therefore, we performed a RIP-PCR assay using the splicing factors that had been previously investigated and found to act as oncogenes in cancer development. We examined SF3B1, U2AF1, and SRSF1, and found that SNORD14E was enriched in the serine and arginine-rich splicing factor 1 (SRSF1) (Fig. 3E). As a member of the serine/arginine (SR) protein family of splicing activators, SRSF1 plays a role in exon definition and exerts exons splicing activation by binding directly to pre-mRNA [19]. After the snoRNA and RNA binding protein form a complex (snoRNPs), biological processes required a directly binding through base-pair interaction between the pre-mRNA and the RNA component of the snoRNP [20]. Next, we investigated the genes with potential SNORD14E-binding sites (IntaRNA program (http://rna.informatik.uni-freiburg.de)) and found three genes (Supplementary Figure A). Subsequently, RIP-PCR was employed to detect the mRNA enriched in SRSF1, and finally, we obtained FOXM1 (Fig. 3F, Supplementary Figure B).

### SRSF1 recruited by SNORD14E recognizes exonic splicing enhancers (ESEs) on VIIa and affects FOXM1 alternative splicing

**Fig. 4:**
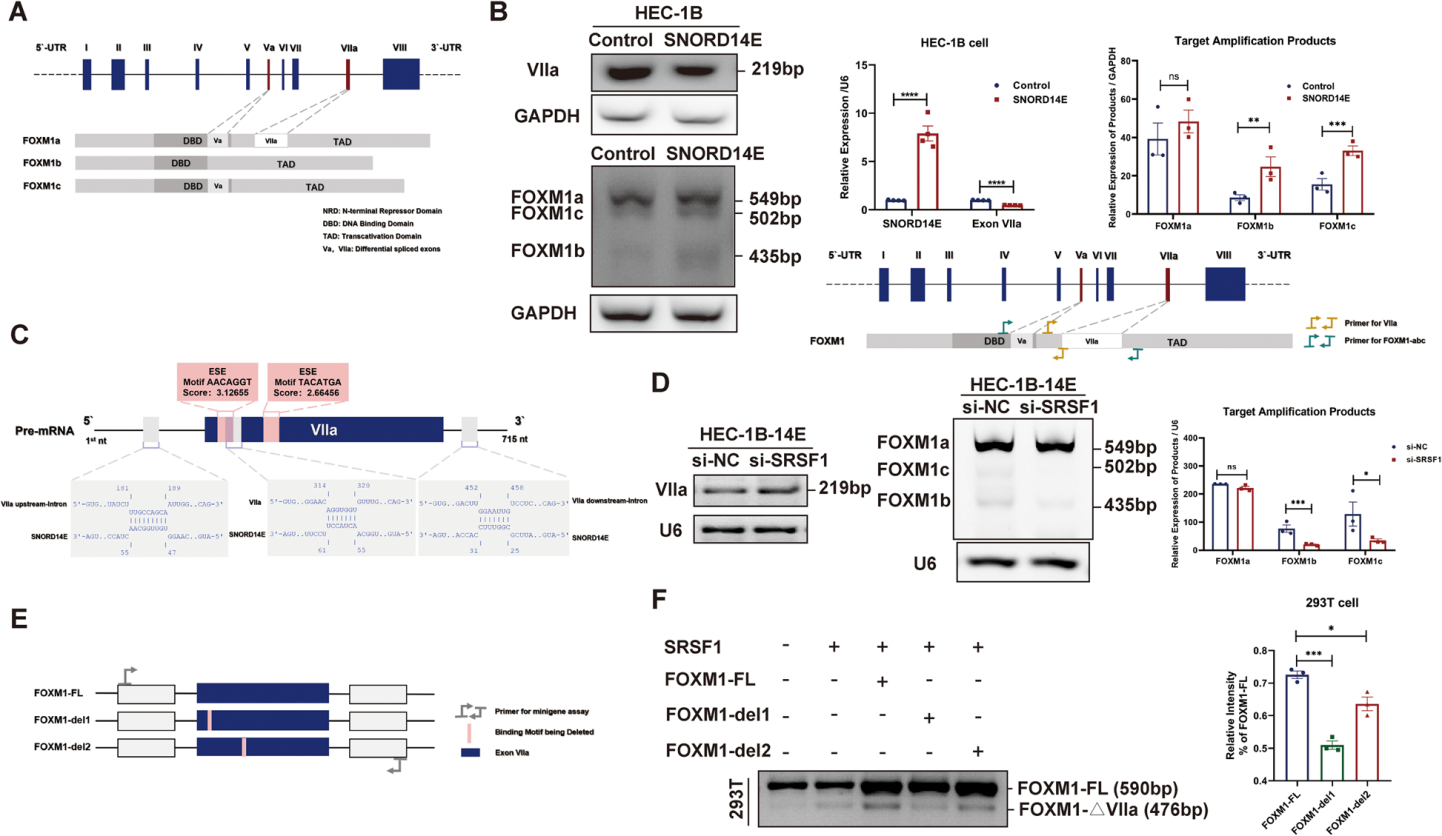
SRSF1 recruited by SNORD14E recognizes exonic splicing enhancers (ESEs) on VIIa and affects FOXM1 alternative splicing. **A** Diagrams of FOXM1 transcripts and its alternative exon Va and VIIa. **B** RT-PCR validation of a selection of alternative splicing events (SE of VIIa caused by SNORD14E). **C** Prediction diagram of ESE and potential base-pair complementary region of SNORD14E. There are ESEs recognized by SRSF1 on exon VIIa, and regions potentially complementary to SNORD14E on the VIIa and flanking introns. **D** HEC-1B cells overexpressing SNORD14E were used. SNORD14E-regulated exon VIIa skip was inhibited by siRNA targeting SRSF1, and the content of the transcripts including exon VIIa (FOXM1b and FOXM1c) were reduced by si-SRSF1. **E** Diagram of FOXM1 minigene deletion mutant plasmids. **F** Splicing analysis of FOXM1 minigene and indicated deletion mutations. The RT-PCR results are quantified and showed in histogram plots. * *P* < 0.05, ** *P* < 0.01, *** *P* < 0.001

FOXM1 consists of ten exons, including exons I-VIII and two alternatively spliced exons (Va, VIIa). Depending on the alternative exons Va and VIIa, three main FOXM1 isoforms can be identified: FOXM1a that includes ten exons, FOXM1b is lacking alternative exons Va and VIIa, and FOXM1c that includes Va but lacks VIIa (Fig. 4A). We constructed primers to determine the abnormal skipped exon of FOXM1 caused by SNORD14E. Then, we amplified all isoforms retaining or skipping the two alternative exons using the primers we designed. As expected, FOXM1 had abnormally skipped exon VIIa in SNORD14E-overexpressing cells, which increased the content of FOXM1b and FOXM1c (Fig. 4B).

Exonic splicing enhancers (ESEs) are binding elements for specific SR proteins which are prevalent and could promote exon definition by directly recruiting the splicing machinery through their RS domain [21–23]. ESE Finder (http://exon.cshl.edu/ESE/) was employed for the prediction of ESEs on VIIa. Two potential ESE elements on VIIa were detected that could bind to SRSF1 (Fig. 4C). Notably, knockdown of SRSF1 significantly inhibited the skipping of FOXM1 VIIa, indicating that the skipping of FOXM1 VIIa was SRSF1-dependent (Fig. 4D).

To obtain more mechanistic insights into SRSF1 regulation of FOXM1 VIIa skipping, a minigene reporter plasmid (FOXM1-FL) was constructed, which was composed of a genomic DNA fragment of the FOXM1 exons VII, VIIa, and VIII and 300 bp sequences at each end of introns. Next, we generated fragment deletion mutation plasmids in which the potential ESEs of exon VIIa were respectively deleted (FOXM1-del1 and FOXM1-del2) based on the FOXM1-FL minigene to explore the function of these ESEs in FOXM1 VIIa skipping (Fig. 4E). FOXM1-del1 and FOXM1-del2 showed lower exon VIIa skipped effects than FOXM1-FL in FOXM1 VIIa skipping (Fig. 4F). Altogether, these results suggested that the selected ESEs could all be recognized by SRSF1, and the recognition might be affected by SNORD14E. Enrichment of the SRSF1-binding motif within FOXM1 VIIa resulted in FOXM1 VIIa skipping, a process that SNORD14E was able to facilitate by recruiting SRSF1.

### SNORD14E induces β-catenin nuclear accumulation and facilitates EC progression through FOXM1 VIIa skipping

**Fig. 5:**
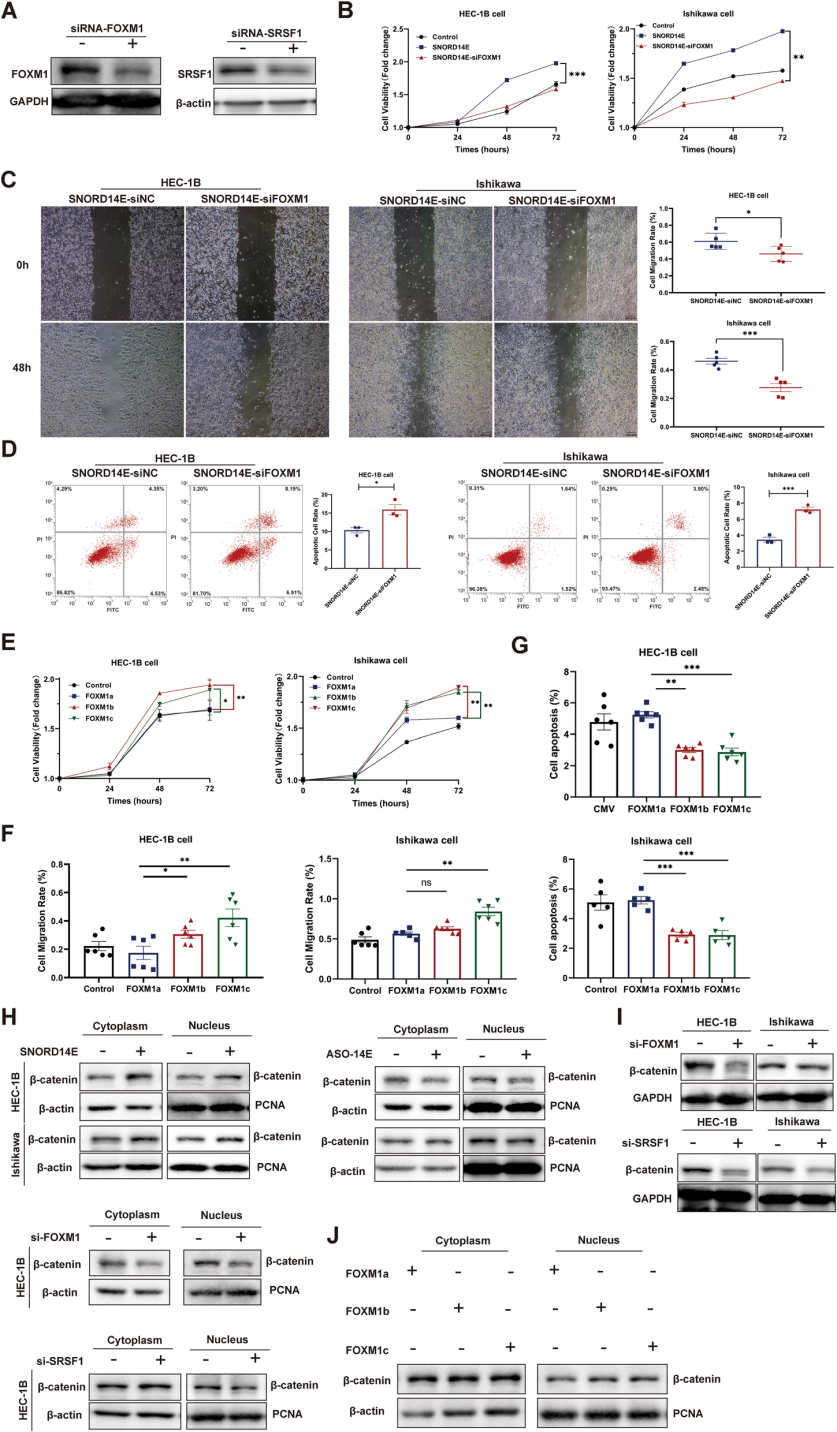
SNORD14E induces β-catenin nuclear accumulation and facilitates EC progression through FOXM1 VIIa skipping. **A** Western-blot of FOXM1 and SRSF1 showing the silencing efficiency of siRNAs against FOXM1 or SRSF1. Cell proliferation (**B**) and quantification of migration and flow apoptosis assays (**C** and **D**) of siRNA targeting FOXM1 (si-FOXM1) treated HEC-1B and Ishikawa cells. **E–G** Plasmids of FOXM1a, FOXM1b and FOXM1c effects on HEC-1B cells and Ishikawa cells. **H** SNORD14E induced nuclear accumulation of β-catenin proteins in HEC-1B cells by western-blot. **I** siRNA targeting FOXM1 or SRSF1 reduced total β-catenin protein in HEC-1B and Ishikawa cells. Knockdown of FOXM1 and SRSF1 leads to a decrease content of β-catenin in the nuclear. **J** FOXM1b and FOXM1c caused more nuclear accumulation of β-catenin protein than FOXM1a

Next, we constructed a siRNA targeting FOXM1 or SRSF1 and determined its inhibitory efficiency (Fig. 5A). The siRNA was transfected into SNORD14E-overexpressing cells, which inhibited EC cell proliferation and migration but increased apoptosis (Fig. 5B–D). The results above indicated that SNORD14E regulated FOXM1 VIIa skipping by recruiting SRSF1, leading to increased FOXM1b and FOXM1c expression. Thus, we further explored whether FOXM1b and FOXM1c promoted the progression of EC. We constructed FOXM1b and FOXM1c plasmids, respectively, and transfected them into Ishikawa and HEC-1B cells. CCK8 analysis showed that the FOXM1b and FOXM1c transfection led to higher cell growth and migration and lower apoptosis than those in the VIIa inclusion isoform FOXM1a (Fig. 5E–G; Supplementary Figure C). In vitro studies revealed that FOXM1b and FOXM1c contributed to tumor growth in EC.

FOXM1 is a transcriptional master regulator of a number of cancers. Hence, the upregulation and activation of FOXM1 can contribute to increased cell proliferation, invasion, metastasis, and angiogenesis. FOXM1 was reported to promote β-catenin nuclear accumulation [24]. In this study, we first detected the expression of β-catenin in both the cytoplasm and the nucleus with SNORD14E overexpression or ASO targeting SORD14E. An accumulation of β-catenin in nuclear was caused by SNORD14E overexpression (Fig. 5H). siRNA targeting FOXM1 and siRNA targeting SRSF1 was separately transfected into cells, which decreased the total β-catenin level and the nucleus β-catenin level after FOXM1or SRSF1 was knocked down. (Fig. 5I). We next constructed plasmids of FOXM1a, FOXM1b, and FOXM1c and transfected them into HEC-1B cells. The results showed that FOXM1b and FOXM1c overexpression led to higher β-catenin expression and nuclear accumulation than that of FOXM1a (Fig. 5J).

### SNORD14E mediates FOXM1 2`-O-methylation modification to increase FOXM1 stability

**Fig. 6:**
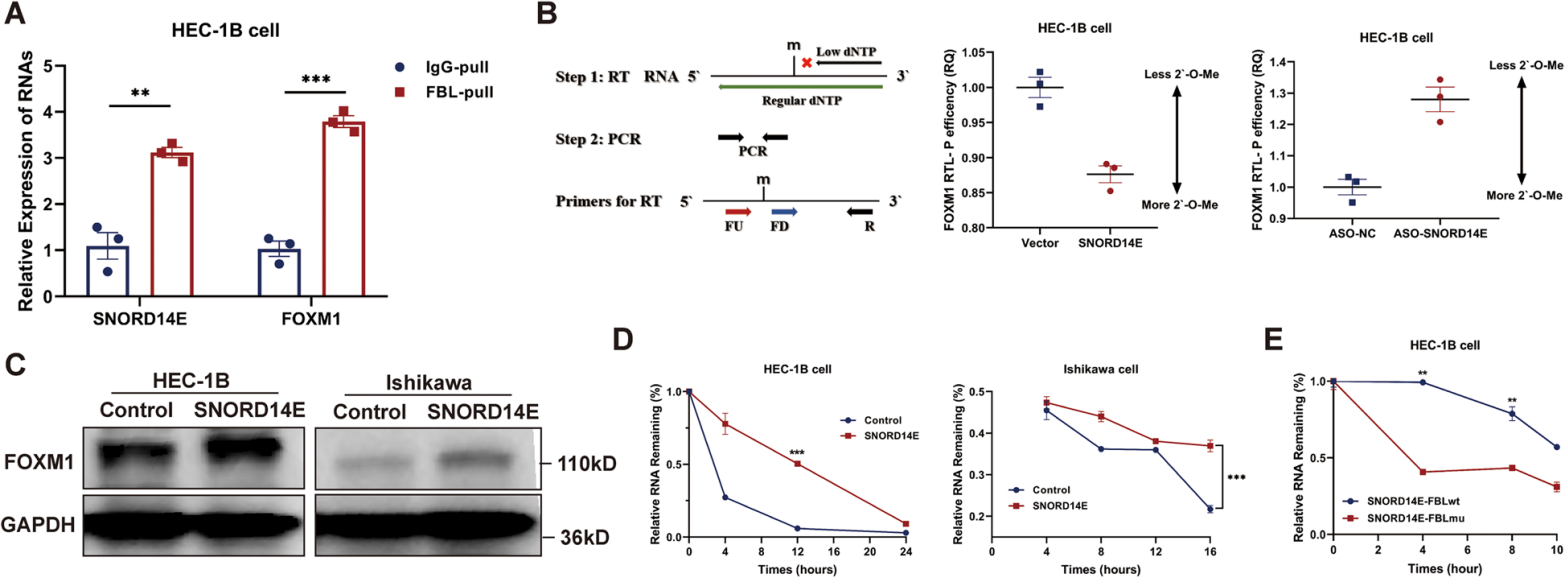
SNORD14E mediates FOXM1 2`-O-methylation modification to increase FOXM1 stability. **A** RIP-PCR showed FOXM1 was enriched on SNORD14E-FBL complex. **B** The FOXM1 mRNA 2`-O-Me activities was increased after SNORD14E overexpression. **C** FOXM1 was upregulated by SNORD14E in EC cells. **D** Promotion of FOXM1 mRNA stability by SNORD14E in HEC-1B cells and Ishikawa cells. **E** FBL-wt and FBL-mu were transfected respectively into HEC-1B cells. The stability of FOXM1 mRNA is reduced by mutating the 2`-O-catalytic region of FBL (FBL-mu). ** *P* < 0.01, *** *P* < 0.001

2`-O-methylation modification of target RNA is the most extensive biological function of SNORDs. As RIP-PCR showed a combination between SNORD14E and the key enzyme 2'-O-methyltransferase fibrillarin (FBL) (Fig. 6A), we next performed Nm-seq to clarify if the recognition of SNORD14E resulted in a 2'-O-methylation modification of FOXM1. Nm-seq results revealed that SNORD14E performed 2`-O-methylation modification on FOXM1 (G909), which was confirmed by RTL-P assay (Fig. 6B). Subsequently, we explored the biological effect of this modification. The Western blot analysis showed that SNORD14E promoted FOXM1 expression (Fig. 6C). After treating the SNORD14E-overexpressing cells with Act-D, we performed RT-PCR to detect the level of FOXM1 mRNA and found that the stability of FOXM1 mRNA in the SNORD14E overexpression group was significantly higher than that in the control group (Fig. 6D). We further constructed FBL-mu that inactivates the key 2`-O-methylation catalytic region of FBL and wild-type FBL-wt as control to eliminate the interference caused by the biological activity of FBL itself. Cells of SNORD14E-overexpressing were transfected with FBL-mu or FBL-wt and treated with Act-D. It showed that FBL without the 2`-O-methylation catalysis counteracted the half-life prolongation of FOXM1 mRNA induced by SNORD14E (Fig. 6E).

### SNORD14E promotes tumor growth in vivo and in PDOs

**Fig. 7:**
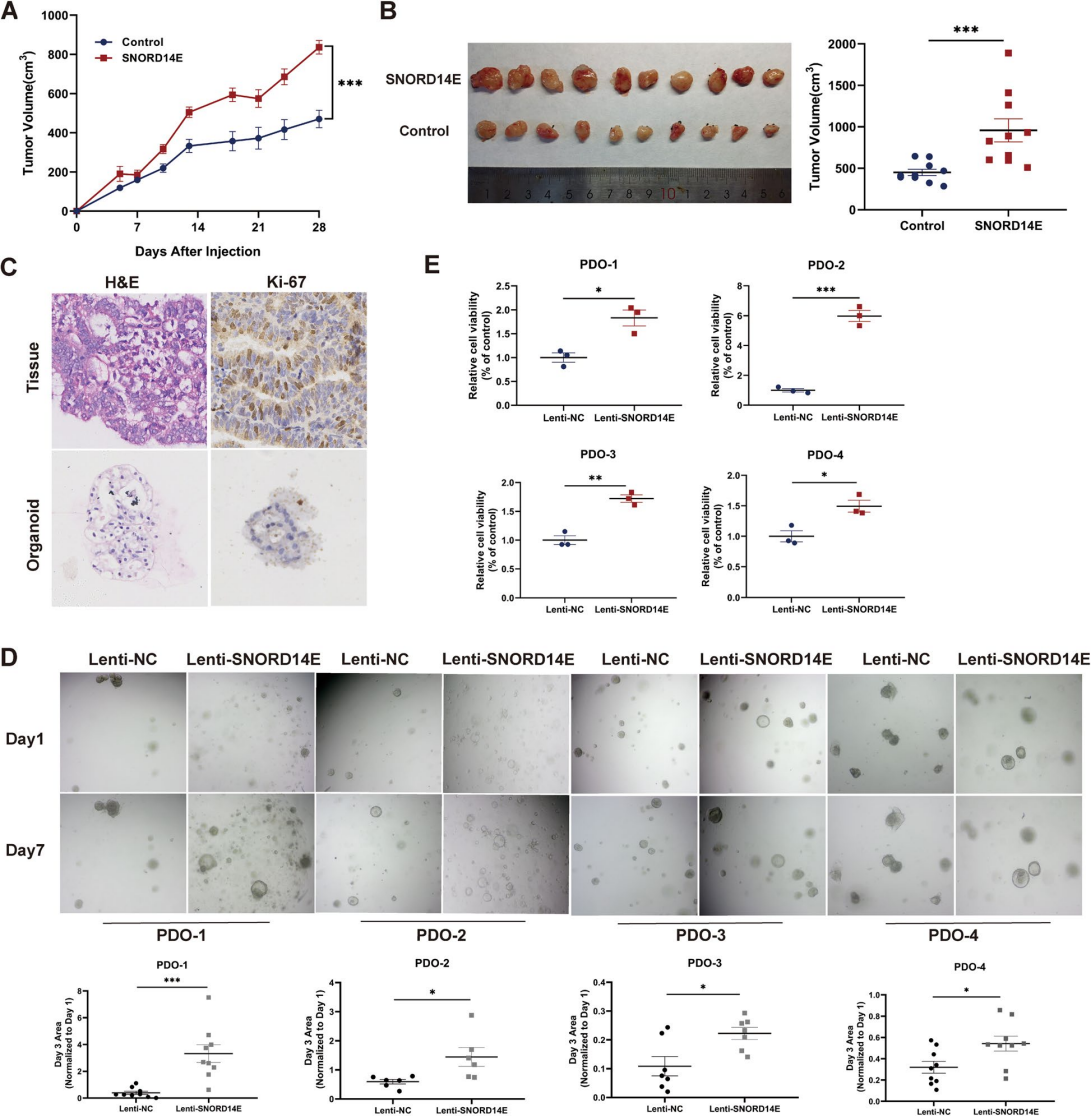
SNORD14E promotes tumor growth in vivo and in PDOs. **A** Xenograft tumor growth were shown for indicated groups. HEC-1B cells were used. **B** Tumor volumes and tumor images of different groups were shown. **C** EC patients-derived organoid (EC-PDO) models were established. Represented images of IHC and HE staining were shown. **D** Images of four EC-PDO were shown and Organoids area was calculated and values were the mean ± SD. of *n* = 9 organoids. **E** Quantification of viability assays following Lentivirus transfection (Lenti-NC and Lenti-SNORD14E) treatment were shown. Cell viability assay was used for detecting affection of SNORD14E on PDO. * *P* < 0.05, ** *P* < 0.01, *** *P* < 0.001

Mouse xenograft experiments were conducted to access the function of SNORD14E in EC progression. HEC-1B cells overexpressing SNORD14E and the corresponding control cells were injected into nude mice subcutaneously. SNORD14E enhanced tumor growth in vivo, as determined by the tumor growth curve (Fig. 7A–B).

EC-PDO models were established that maintained the histological and molecular features of parental EC (Fig. 7C). EC tissue was digested into 3–10-cell clusters, transfected with SNORD14E lentivirus or control lentivirus, and seeded into 3D culture media. The SNORD14E transfection resulted in a greater organoid diameter than that in the control group (Fig. 7D). Cell Counting-Lite® 3D Luminescent Cell Viability Assay was used for detecting PDO viability (Fig. 7E).

### SNORD14E is a potential therapeutic target for EC

**Fig. 8:**
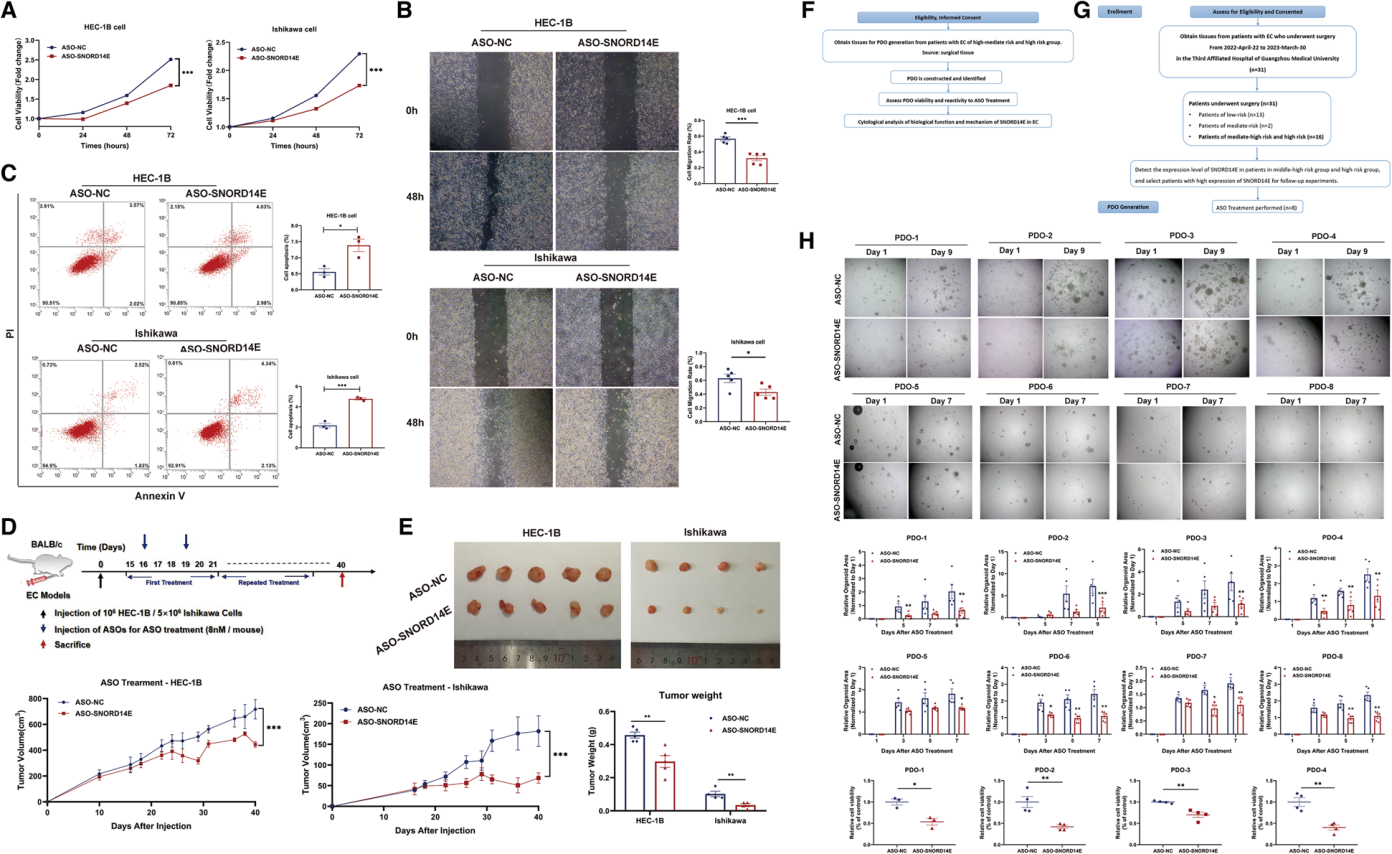
SNORD14E is a potential therapeutic target for EC. **A-C** ASO targeting SNORD14E inhibited cell proliferation, migration and promoted cell apoptosis through CCK-8 assay, scratch assay and flow apoptosis assay. **D** Schematic illustration of subcutaneous injection of cells and ASO treatment on nude mice. **E** HEC-1B cell group (left) and Ishikawa cell group (right) were treated with ASO-NC or ASO-SNORD14E separately, and images of indicated groups, tumor volume and tumor weight were shown. **F** Study schema. **G** Graphical flow-chart of the study. **H** Representative images of the eight EC-PDOs treated with ASO-SNORD14E or ASO-NC and quantification of organoid size were shown. Cell viability was detected by the CellCounting-Lite 3D luminescent cell vitality and normalized with ASO-NC. * *P* < 0.05, ** *P* < 0.01, *** *P* < 0.001

Recently, ASO drugs have been considered as therapeutic agents for EC as they are able to target RNAs more precisely than small-molecule compounds, which has been verified in vivo and in vitro. Based on the aforementioned results, we hypothesized that SNORD14E might be of value in the treatment of EC patients. First, we used ASO to knockdown the expression of SNORD14E, which significantly reduced the cell proliferation, migration, and invasion abilities, whereas the apoptosis was increased (Fig. 8A–C). Then, a xenograft mouse model was established to evaluate the therapeutic efficacy of ASO-SNORD14E treatment. A 5'-end cholesterol modification and a conventional phosphodiester-linked ASO were employed to achieve higher in vivo activity. HEC-1B and Ishikawa cells were respectively inoculated subcutaneously in nude mice, and mice with the same cells were randomly divided into two groups (ASO-NC and ASO-SNORD14E) two weeks later, when the tumor size reached 50–100 mm^3^. The therapeutic is presented in Fig. 8D. The tumor growth curve shows that the SNORD14E depletion by in vivo-optimized SNORD14E inhibitor (ASO-14E) application decreased the growth of the tumors in both HEC-1B and Ishikawa cell xenografts (Fig. 8E).

Encouraged by the efficacy of the selected ASO to inhibit SNORD14E and in view of the important clinical implications of such results, we generated PDO models and performed transfection-free ASO-mediated knockdown experiments in the three PDO models. The study design is showed in Fig. 8F and 8 cases of EC-PDO models were established (Fig. 8G, Supplementary Table 4). Consistent with the results obtained in the xenograft models, the growth of the PDO models in the ASO-14E group was significantly weaker than that in the control group (Fig. 8H). Collectively, the above results suggest that SNORD14E could be a potential target for EC treatment.

## Discussion

Patients with advanced and recurrent EC have poor prognosis, and the 5-year survival rate is less than 20% [3]. Recognizing the patients with high-risk factors and giving them the best adjuvant therapy is therefore of critical importance to improve the management of the disease. At present, there are still deficiencies in biomolecular classifications including the initial TCGA biomolecular classification, ProMisE, TransPORTEC, Parra-Herran and so on, and no agreement has been reached. To find valuable therapeutic targets for patients with EC, we screened data obtained from the TCGA database and found that SNORD14E was overexpressed in EC tissues. and patients with high level of SNORD14E were distributed in the TCGA biomolecular classification subgroups without difference. These results suggest that SNORD14E may be a valuable target for prognosis independent of TCGA biomolecular classification. In the in vitro experiments we conducted, SNORD14E promoted the proliferation, migration, and invasion of endometrial carcinoma cells and acted as an oncogene in EC. Next, we constructed the targeted drug ASO-SNORD14E and found that ASO-SNORD14E could significantly inhibit cell proliferation and promote cell apoptosis. TCGA-UCEC database indicated that high expression of SNORD14E caused poor prognosis in EC patients. Based on the ESMO-ESGO-ESTRO prognostic risk stratification, the proportion of patients with high level of SNORD14E in the middle-high risk group and high-risk group was more than 50%.

In recent years, new molecular targeted therapies have achieved significant efficacy in clinical practice. Expanding the existing understanding of the molecular mechanism of EC may promote the development of effective targeted therapies and improve the overall prognosis of patients with EC. As a functional model, PDOs contain the multi omics characteristics of the original tumor and simulate the response of patients to targeted drugs, which to some extent guide clinical treatment, and are widely concerned. In the study, we successfully constructed middle-high risk and high-risk EC-PDO models and verified the therapeutic value of ASO-SNORD14E from xenograft model to preclinical PDO assay. Above all, ASO targeting SNORD14E has a therapeutic effect on EC-PDO model which derived from patients of high level of SNORD14E in the middle-high risk group and high-risk group. The results of this study provide a new option for the targeted therapy of about 50% of the patients with high expression of SNORD14E in the middle-high risk and high-risk group of EC. ASO targeting SNORD14E may be an effective treatment for EC.

To further explore the regulatory mechanism of SNORD14E, we analyzed our RNA-seq results and found that SNORD14E caused Alternative splicing events of a large number of tumor-related genes. Dysregulation of splicing events can change protein function and lead to cancer initiation and progression [25–28]. In this study, RIP-PCR results confirmed the binding of the splicing factor from the SR protein family called splice factor 1 (SRSF1) to SNORD14E. Subsequently, we screened the genes involved in AS events in the RNA-seq results, employed bioinformatics analysis, and finally focused on FOXM1 among EC-related genes because it included binding sites for SNORD14E and was enriched in SRSF1.

FOXM1 has become a oncoprotein and a powerful biomarker of poor prognosis for many human malignancies in recent studies [29]. The upregulation and activation of FOXM1 can lead to a variety of tumor-associated phenotypes such as cell proliferation, tumor stem cells, drug resistance, invasion, metastasis, and angiogenesis [30–33], which have been reported as top-level gene expression biomarkers with poor prognosis [34]. The high FOXM1 expression in endometrial carcinoma is closely associated with the prognosis, pathological stage, and clinical grade of endometrial carcinoma patients [35] and can thus serve as a marker of endometrial carcinoma prognosis and a candidate target for its treatment [36]. The FOXM1 gene comprises ten exons, including exons I-VIII and the alternatively spliced exons Va and VIIa [37–39]. Based on the AS of exons Va and VIIa, three FOXM1 subtypes can be classified: FOXM1a including all ten exons, FOXM1b with lacking alternative exons Va and VIIa, and FOXM1c including the alternative exon Va but lacking the alternative exon VIIa [39, 40]. Our results indicate that the overexpression of SNORD14E promotes the exon VIIa skipping of FOXM1, resulting in an increase in the transcript FOXM1b and FOXM1c subtypes.

AS is characterized by the ability to accurately identify and splice the correct splice sites among numerous potential splice sites [41]. The proper recognition of the splice sites is ensured by high degrees of matching to the consensus sequences or by the assistance of cis-acting elements and trans-acting factors [42, 43]. Reportedly, exon skipping may be affected by cis-acting elements such as exon splice enhancers (ESEs) [23, 42]. Studies have shown that SRSF1 usually binds to ESEs to promote splicing [23]. The AS event mediated by SRSF1 and the carcinogenic effects of SRSF1 have been observed in several tumors [44–46]. For example, the splice switch of MYO1B directly regulated by SRSF1 increased the carcinogenic potential of glioma cells through the PDK1/AKT and PAK/LIMK pathways [47]. Next, to explore the effect of SNORD14E on the exon skipping of FOXM1 VIIa, we predicted the ESEs element located on THE alternative exon VIIa using the splice site prediction program ESE Finder (http://exon.cshl.edu/ESE/) [48, 49], suggesting that two potential ESE elements on the alternative exon VIIa might bind to SRSF1. We found that SRSF1 always led to the exclusion of the optional exon VIIa of FOXM1, which was more pronounced in cells with SNORD14E overexpression. The effect of SNORD14E on the FOXM1 exon VIIa skipping was rescued by knocking down SRSF1 in endometrial carcinoma cell lines, suggesting that SNORD14E recognized FOXM1 and promoted the FOXM1 exon VIIa skipping by recruiting SRSF1 in combination with SRSF1. Minigene analysis showed that the enrichment of the SRSF1-binding motif within exon VIIa resulted in exon VIIa skipping, whereas SNORD14E was able to facilitate this process by recruiting SRSF1.

Of note, the assessment of the functional characteristics indicates that FOXM1b and FOXM1c are transcriptionally active, whereas FOXM1a is not [50–53]. Both FOXM1b and FOXM1c can promote malignant behavior of tumors, of which, FOXM1c may promote a proliferation and metastasis phenotype, whereas FOXM1b induces more pronouncedly a migration and invasion phenotype [54]. After the plasmids of three FOXM1 subtypes were constructed and tested in vitro, in contrast to FOXM1a, FOXM1b and FOXM1c were found to be able to promote the proliferation and migration of EC cells and inhibit apoptosis. However, after the transfection of siRNA to knock down FOXM1, the pro-cancer effect of SNORD14E on EC cells was almost completely offset.

Recent snoRNA research has been focused mainly on the 2 '-O-methylation modification function. That is, C/D box snoRNA performs 2 '-O- methylation modification in a variety of RNAs, including mRNA, through base complementary pairing and is involved in gene regulation [55, 56]. Nm (2 ′-O-methylation, where N represents any nucleotide) is one of the most common and widely distributed RNA post-transcriptional modifications in RNA. Some of these modifications are directed by box C/D snoRNAs and catalyzed by FBL [57]. Using RIP detection, we found that SNORD14E was connected to FBL, indicating that SNORD14E might have a Nm modification. Nm-seq results suggested that SNORD14E was capable of NM modification of multiple genes, including FOXM1. RTL-P assay confirmed the Nm modification of FOXM1 caused by SNORD14E. Notably, the Nm modification prolonged the half-life of FOXM1 mRNA and inhibited the protein degradation of FOXM1. We further mutated FBL as the domain of the key enzyme for 2'-O-methylation modification and found that, relative to FBL-wt, FBL-mu without the 2'-O-methylation modification could not affect the degradation of FOXM1 mRNA and protein, *i.e*., the effect of SNORD14E on mRNA and protein levels of FOXM1 was achieved after Nm modification.

β-catenin is involved in a range of tumor-related signaling pathways and is tightly regulated at three levels: protein stability, subcellular localization, and transcriptional activity [58, 59]. FOXM1 enhances the nuclear localization of β-catenin and the expression of downstream target genes by binding to β-catenin to promote the occurrence and development of a variety of tumors. In gliomas, FOXM1 promotes tumorigenesis by direct interaction with β-catenin and acts as a cofactor for β-catenin stabilization [24]. However, the effects of the interactions between FOXM1 and β-catenin in EC have not been elucidated. In this study, we discovered that SNORD14E promoted the nuclear aggregation of β-catenin which was inhibited after the knockdown of FOXM1. In the analysis of the effect of SNORD14E on alternative FOXM1 splicing, we found that the knockdown of SRSF1 also inhibited the nuclear aggregation of β-catenin. Further, we performed analyses to determine the nuclear content of β-catenin after the overexpression of FOXM1a, FOXM1b, and FOXM1c, respectively. We established that both FOXM1b and FOXM1c promoted more the nuclear aggregation of β-catenin than FOXM1a. The aforementioned results indicated that SNORD14E could promote the nuclear aggregation of β-catenin through multi-faceted regulation of FOXM1, thus promoting the occurrence and development of endometrial carcinoma.

## Conclusions

In this study, we found that SNORD14E is highly expressed in EC and reduces DFS and RFS of patients with EC. SNORD14E can promote the malignant phenotype of EC cells. We further clarified the mechanism of SNORD14E in EC, and confirmed the application prospect of ASO drugs targeting SNORD14E in patients with middle-high risk and high-risk prognostic factors of EC.

## Limitations

This study is a preclinical research based on PDOs. PDOs can better simulate the characteristics of molecular properties and tumor heterogeneity of parental tumor tissues. They are suitable models for preclinical research of drugs and closer to the true reflection of patients on drugs. Nevertheless, the results of preclinical research need to go through procedures including pharmacology, pharmacokinetics, toxicology, long-term toxicity tests and phase I-III trials of clinical research before they can be finally applied to patients. There is still a long way to go from this study to clinical application.

### Supplementary Information

**Additional file 1:** **Supplementary Figure A.** There were binding region between SNORD14E and MELK and CCDC150. **Supplementary Figure B. **The mRNAs of MELK and CCDC150 were not enriched on SRSF1. **Supplementary Figure C.** FOXM1b and FOXM1c promoted cell migration and inhibited cell apoptosis comparing with FOXM1a and Control.

**Additional file 2:** **Supplementary Table 1. **Primer sequences used for PCR assays. **Supplementary Table 2. **Primer sequences used for RTL-P assays. **Supplementary Table 3. **The oligonucleotides used in this study. **Supplementary Table 4. **Patient clinical characteristic for the patient-derived cancer organoids.

**Additional file 3: Supplementary Table 5.** The full sequence of different isoforms of FOXM1.

**Additional file 4:** **Supplementary Table 6.** Sequences of plasmids constructed for minigene assay.

## Data Availability

The resources, tools and codes used in our analyses were described in each method section in the methods. For any further of the data requests, please contact the corresponding author. The public datasets of TCGA analysed during the current study is: TCGA-UCEC https://portal.gdc.cancer.gov/projects/TCGA-UCEC.

## Abbreviations

TCGA: The Cancer Genome Atlas
PDO: Patient-derived organoid
qRT-PCR: Real-time quantitative reverse transcription-polymerase chain reaction
RIP: RNA immunoprecipitation
AS: Alternative splicing
ASO: Antisense oligonucleotide
IHC: Immunohistochemistry
IF: Immunofluorescence
Nm-seq: 2’-O-Methylation Modification sequencing
RTL-P: Reverse Transcription at Low deoxy-ribonucleoside triphosphate concentrations followed by PCR
