# Supplementary material for: A patient-derived organoid-based study identified an ASO targeting SNORD14E for endometrial cancer through reducing aberrant FOXM1 Expression and β-catenin nuclear accumulation
Source: J Exp Clin Cancer Res. 2023 Sep 5;42:230. doi: 10.1186/s13046-023-02801-2 (PMC10478245; doi:10.1186/s13046-023-02801-2)
Supplement: Supplementary file 1 — Additional file 1: Supplementary Figure A. There were binding region between SNORD14E and MELK and CCDC150. Supplementary Figure B. The mRNAs of MELK and CCDC150 were not enriched on SRSF1. Supplementary Figure C. FOXM1b and FOXM1c promoted cell migration and inhibited cell apoptosis comparing with FOXM1a and Control. [file 13046_2023_2801_MOESM1_ESM.docx]

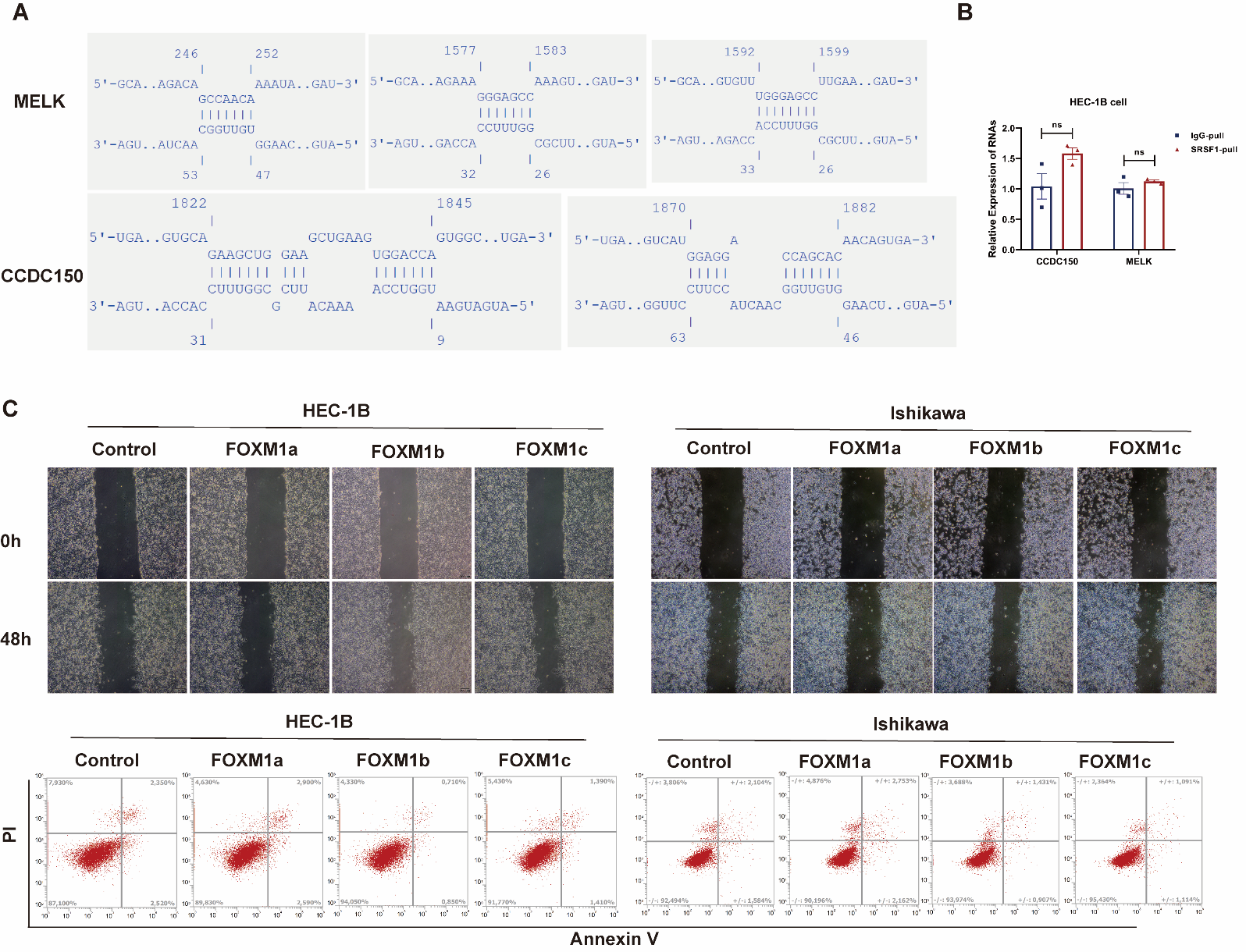


**Supplementary Figure A** There were binding region between SNORD14E and MELK and CCDC150.

**Supplementary Figure B** The mRNAs of MELK and CCDC150 were not enriched on SRSF1.


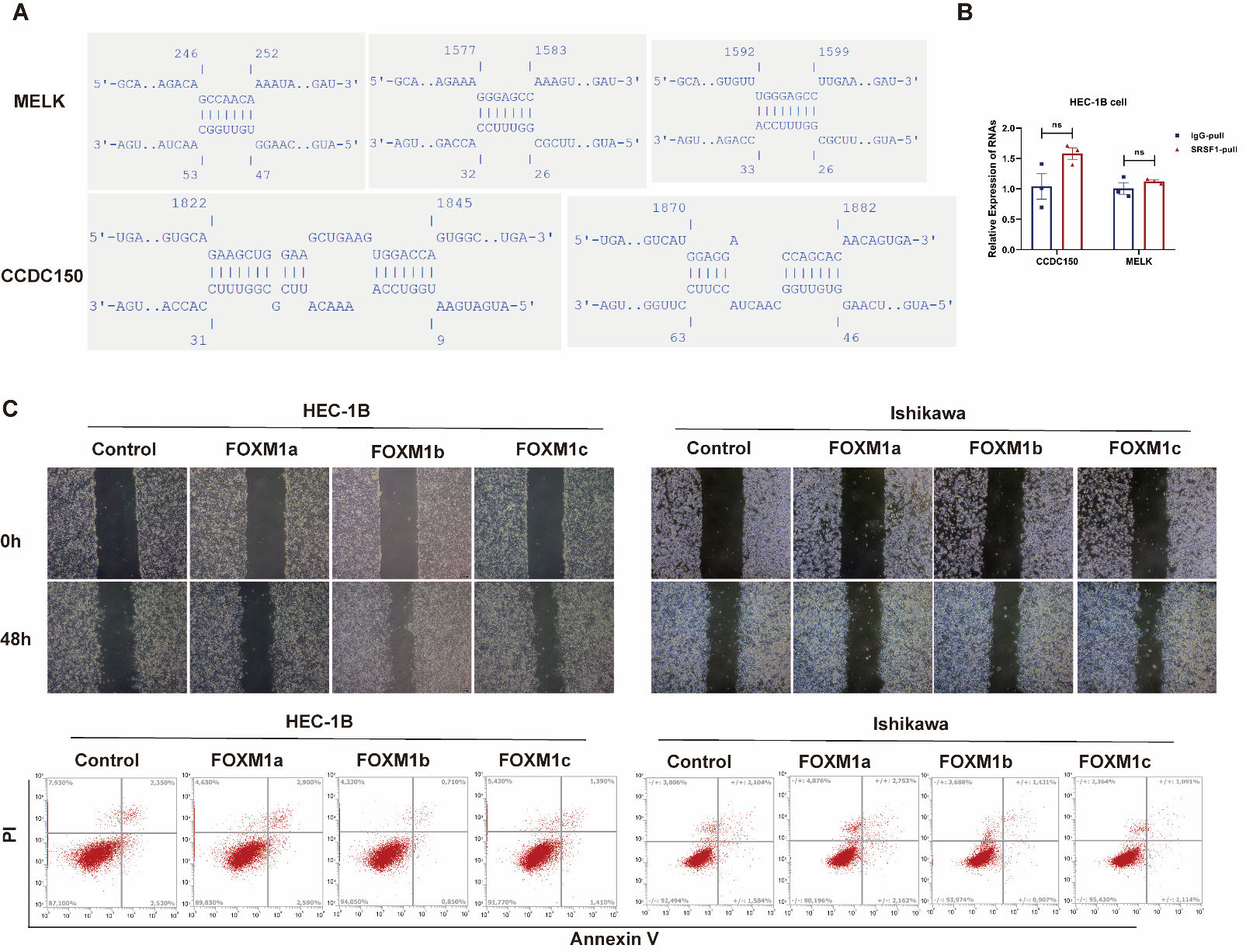


**Supplementary Figure C** FOXM1b and FOXM1c promoted cell migration and inhibited cell apoptosis comparing with FOXM1a and Control.
