# Supplementary material for: A patient-derived organoid-based study identified an ASO targeting SNORD14E for endometrial cancer through reducing aberrant FOXM1 Expression and β-catenin nuclear accumulation
Source: J Exp Clin Cancer Res. 2023 Sep 5;42:230. doi: 10.1186/s13046-023-02801-2 (PMC10478245; doi:10.1186/s13046-023-02801-2)
Supplement: Supplementary file 2 — Additional file 2: Supplementary Table 1. Primer sequences used for PCR assays. Supplementary Table 2. Primer sequences used for RTL-P assays. Supplementary Table 3. The oligonucleotides used in this study. Supplementary Table 4. Patient clinical characteristic for the patient-derived cancer organoids. [file 13046_2023_2801_MOESM2_ESM.docx]

**Supplementary Table.1** Primer sequences used for PCR assays

|  | **Forward primer** | **Reverse primer** |
| --- | --- | --- |
| **SNORD14E** | 5'-GATGAATGGTCCAAAACA-3' | 5'-ACATCCAAGGAAGGTAGTT-3' |
| **FOXM1-Total** | 5`-ATACGTGGATTGAGGACCACT-3` | 5’-TCCAATGTCAAGTAGCGGTTG-3’ |
| **FOXM1-VIIa** | 5'-GAAGCCACTGCTACCACG-3' | 5'-TCGCCACTAAAGAACTTACTCAT-3' |
| **FOXM1-abc** | 5`-AGAACTCCATCCGCCACA-3` | 5`-TCGCCACTAAAGAACTTACTCAT-3` |
| **FOXM1-minigene** | 5`-GAAGATGAAGCCACTGCTACCAC-3` | 5`-CTCCTCTCCCTGTGTTGAATCAC-3` |
| **U6** | 5`-CTCGCTTCGGCAGCACA-3' | 5'-AACGCTTCACGAATTTGCGT-3' |
| **GAPDH** | 5'-CCCATCACCATCTTCCAGGAG-3' | 5'-GTTGTCATGGATGACCTTGGC-3' |

All of the primers are synthesized by BGI Genome company.

**Supplementary Table.2** Primer sequences used for RTL-P assays

|  | **Forward primer** | **Reverse primer** |
| --- | --- | --- |
| **FOXM1-in** | 5'-TTGCGAGCAGAAACGGG-3' | 5'-GGCACTGGGGTGAATGG-3' |
| **FOXM1-out** | 5`-TGGAGCAGCGACAGGTTA-3` | 5’-GGCACTGGGGTGAATGG-3’ |

All of the primers are synthesized by BGI Genome company.

**Supplementary Table. 3** The oligonucleotides used in this study

|  | **Sequences** |
| --- | --- |
| **ASO-SNORD14E** | TCCAAAACATTCGCGGTTTC |
| **SiRNA FBL** | GGGCTAAGGTTCTCTACCT |
| **SiRNA SRSF1** | CGACGGCTATGATTACGAT |
| **SiRNA FOXM1** | CCAACAATGCTAATATTCA |

All of the siRNAs are synthesized by Ribobio Company.

**Supplementary Table. 4** Patient clinical characteristic for the patient-derived cancer organoids
